# The Association between a Novel Baby-Friendly Hospital Program and Equitable Support for Breastfeeding in Vietnam

**DOI:** 10.3390/ijerph18136706

**Published:** 2021-06-22

**Authors:** Caroline M. Joyce, Sherry Shu-Yeu Hou, Binh T. T. Ta, Duong Hoang Vu, Roger Mathisen, Ilona Vincent, Vinh Nguyen Duc, Arijit Nandi

**Affiliations:** 1 Department of Epidemiology, Biostatistics, and Occupational Health, McGill University, Montreal, QC H3A 1A3, Canada; sherry.hou@mail.mcgill.ca (S.S.-Y.H.); arijit.nandi@mcgill.ca (A.N.); 2 Alive & Thrive Southeast Asia, FHI Solutions, Hanoi 100000, Vietnam; tbinh@fhi360.org (B.T.T.T.); vduong@fhi360.org (D.H.V.); rmathisen@fhi360.org (R.M.); 3 Institute for Health and Social Policy, McGill University, Montreal, QC H3A 1A3, Canada; ilona.vincent@mcgill.ca; 4 Ministry of Health, Hanoi 100000, Vietnam; ndvinh_moh@yahoo.com

**Keywords:** breastfeeding, infant health, child health, healthcare program evaluation, government public health partnership, equitable access to breastfeeding, baby-friendly hospital initiative

## Abstract

*Background:* Rates of early initiation of breastfeeding are low in Southeast Asia, despite evidence that increased initiation of early breastfeeding would lead to better long-term infant and child health and decrease inequities in long-term health and well-being. In response, a novel performance-based, baby-friendly hospital program designates hospitals that adhere to evidence-based early essential newborn care (EENC) and breastfeeding interventions as Centers of Excellence for Breastfeeding (COE). This study examined whether hospital participation in the program was associated with better breastfeeding outcomes. *Methods:* Hospitals (*n* = 28) were invited into the program in December 2018. Hospitals developed an improvement plan for promoting a breastfeeding-friendly environment and meeting the standards of the COE accreditation process and were enrolled on a rolling basis over the course of a year. Post-partum surveys were conducted with parents (*n* = 9585) from January 2019 through April 2020 to assess their breastfeeding and post-partum experience. Segmented regression models were used to assess how breastfeeding outcomes evolved before and after hospital enrollment in the COE program. *Results:* Enrollment was associated with a 6 percentage-point (95% CI: 3, 9) increase in the level of early initiation of breastfeeding, which continued to increase in the post-enrollment period, and a 5 percentage-point (95% CI: 2, 9) increase in the level of exclusive breastfeeding during hospital stay. We did not observe evidence that enrollment was immediately associated with receipt of lactation counseling or exclusive breastfeeding at survey time. *Conclusion:* The prevalence of early and exclusive breastfeeding increased after enrollment in the COE program, suggesting that the program has the potential to improve breastfeeding initiation rates and longer-term child health and well-being. Further research should be conducted to examine whether the program has an impact on the overall duration of breastfeeding.

## 1. Introduction

Breastfeeding is an evidence-based intervention for promoting children’s health, development, and survival [1,2,3,4,5,6]. The provision of mother’s breastmilk to infants within one and a half hours of birth, referred to as early initiation of breastfeeding (EIBF), is associated with continued exclusive breastfeeding (EBF) at discharge and through the first 6 months of life [7,8,9,10]. A 2017 systematic review showed that infants who initiated breastfeeding more than 24 h after birth had a twofold greater risk of neonatal mortality compared to infants who initiated breastfeeding within one hour [11]. Nonetheless, children in many parts of the world are not breastfed according to the World Health Organization (WHO) and United Nations Children’s Fund (UNICEF) recommendations, which include breastfeeding initiation within one and a half hours of birth and EBF for the first six months [6,12]. EIBF remains low, ranging from 41% in South Asia to 45% in Sub-Saharan Africa [13], and only 37% of children under 6 months (i.e., 0–6 months) are exclusively breastfed in low- and middle-income countries (LMICs) [6]. Using the new *Cost of Not Breastfeeding Tool*, “595,379 childhood deaths from diarrhea and pneumonia each year can be attributed to not breastfeeding according to global recommendations from WHO and UNICEF”, estimated to amount to USD 341.3 billion in total economic loss [14].

Previous research has emphasized the importance of breastfeeding in reducing the risk of disease, not only for common illnesses that occur in infancy, but also for rarer and serious illnesses. The rates of hospitalizations for pneumonia and severe lower respiratory tract infection are higher among non-breastfed infants than among those are breastfed. A meta-analysis found a significant inverse association between breastfeeding and sudden infant death syndrome (SIDS) [15].

There is substantial evidence on the effectiveness of specific interventions to support EIBF, such as encouraging early and uninterrupted skin-to-skin contact between mothers and infants shortly after birth [16,17,18,19]. Many of these simple, evidence-based practices have been codified by the WHO’s *Ten Steps to Successful Breastfeeding* and as Early Essential Newborn Care (EENC) practices for preventing neonatal morbidity and mortality [20]. The launch of the Baby Friendly Hospital Initiative (BFHI) in 1991 aimed to institutionalize the WHO *Ten Steps* on a global scale. If implemented effectively, BFHI has the potential to influence breastfeeding practices [21,22,23]. At the population level, however, the coverage of BFHI interventions remains low [6]. Although an estimated 71% of countries have an operational BFHI program, only 10% of newborns globally were born in a facility that could document its full adherence to the *Ten Steps* [24]. In addition to poor coverage, sustaining BFHI standards has been a challenge due to inadequate funding, monitoring, and re-evaluation [24,25]. This gap between research and practice reflects our limited understanding of the science of scaling-up breastfeeding programs, leading to calls to identify, implement, and evaluate the effectiveness of novel strategies to ameliorate the country-level implementation of BFHI, with the goal of reaching 100% of maternity hospitals and sustaining standards over time [26].

Vietnam provides an ideal context for addressing this knowledge–practice gap. In 2016, the prevalence of EBF within the first 6 months in Vietnam was 24.3%, which ranks it near the bottom among Southeast Asian countries [20]. Additionally, according to the UNICEF Multiple Indicator Cluster Surveys (MICS), the prevalence of EIBF dropped from 60% to 40% between 2006 and 2011 [27], and by 2014, only 27% of infants were breastfed within an hour of childbirth [28]. Increases in the marketing of breastmilk substitutes in LMICs, as well as frequent violations of the World Health Assembly’s (WHA) International Code of Marketing of Breast-milk Substitutes (ICMBS), have encouraged the substitution of breastfeeding by formula for infants and young children [29,30]. Rates of institutional birth by cesarean section have also increased in LMICs, including Vietnam [28,31,32]. This increase may have lowered EIBF, since newborns delivered via cesarean are more likely to be separated from mothers immediately after birth. Cesarean childbirth may also hinder the “rooming-in” of the infant and immediate skin-to-skin contact, which also leads to lower rates of EIBF [33,34,35].

To address these trends, Vietnam has been the target of a concerted, multisectoral effort to scale-up evidence-based interventions to improve breastfeeding practices. Alive & Thrive (A&T), a global nutrition initiative managed by FHI Solutions, in partnership with Vietnam’s Ministry of Health (MOH), developed a performance-based model for BFHI—the Centers of Excellence for Breastfeeding (COE)—to advance nationwide implementation and sustained adherence to evidence-based EENC and breastfeeding interventions in maternity hospitals in response to updated BFHI guidelines [20]. This study examined whether enrollment in the COE model was associated with EIBF, EBF during hospital stay, EBF after discharge at the time of survey, and receipt of lactation counseling during hospital stay using data from a pilot study conducted in 28 hospitals in 2019–2020. Additionally, to assess equitable implementation of the program, we investigated whether these associations varied by type of birth (vaginal or cesarean).

## 2. Materials and Methods

### 2.1. Intervention and Study Sample

The pilot program began in December 2018, when hospitals were invited by the MOH and A&T to enroll in the COE program, which was the intervention of interest. Hospitals were enrolled in this pilot program on a rolling basis over the span of a year, after which all surveyed hospitals were enrolled in the program. Hospital enrollment date was defined as the date of first coaching session with A&T. When a hospital enrolled in the program, it was provided with technical assistance from A&T and the MOH to develop an improvement plan for promoting a breastfeeding-friendly environment and meeting the standards of the accreditation process. As part of this planning process, phone surveys with mothers after discharge and an in-hospital evaluation were conducted by the provincial Center for Disease Control (CDC) and/or provincial Department of Health (DOH) and/or MOH officials to assess the hospital’s early newborn care and breastfeeding environment. Results from the phone surveys with mothers were then provided quarterly to hospitals. They were also provided with an improvement plan developed in collaboration with A&T to help hospitals meet the independent qualification for supportive supervision of EENC, adhere to the *Ten Steps*, and comply with the WHA International Code of Marketing of Breast-milk Substitutes, according to WHO and MOH guidelines. Details regarding the accreditation process are provided in Appendix A.

The data for this study come from post-discharge phone surveys of mothers of infants born from 28 participating hospitals. Surveys began in January 2019 and continued until the end of the study period for these analyses in April 2020. As such, surveys included infants who were born before and after each hospital enrolled, which provides pre-intervention and post-intervention data. Hospitals submitted lists of mothers and infants recently discharged, with separate lists for vaginal vs. cesarean section births. Both pre-term (<37 weeks) and full-term (≥37 weeks) infants were included in these lists. From this, the MOH/DOH randomly selected numbers to call. Mothers from the preceding quarter were called between 10 and 45 days after discharge from the hospital. If the mother did not answer the phone, the person who did was asked if they were able to answer the questions relating to childbirth and hospital stay.

A breakdown of surveyed births by hospital, along with hospital enrollment dates, can be seen in Appendix B. The unit of enrollment and analysis was the birth, with data collected via surveying the mother. The rate of births by cesarean section was chosen to reflect Vietnam’s national cesarean section rate [36]. Respondents were asked 13 questions relating to the mother’s and infant’s experience with breastfeeding support in the hospital (Appendix C). Questions were developed in consultation with MOH, Learning & Research Center for Newborn Care and Human Milk, which based the questions on WHO guidelines [37,38]. Births were defined as occurring in an enrolled hospital (the intervention) if the hospital had completed at least one accreditation training session. The McGill University Faculty of Medicine Institutional Review Board approved the study. For a more detailed description of the accreditation and interview processes, please refer to external documentation [39,40].

### 2.2. Outcomes

Study outcomes were self-reported survey responses about the birth and newborn care experience. Three outcomes were derived from binary yes/no responses to questions about the in-hospital experience. An infant was coded as receiving EIBF if the mother answered “yes” to both of these questions: “Was your child breastfed within 90 min after birth?” and “Was your baby in skin-skin contact with you for at least 90 min?” EBF while in the hospital was coded if the mother responded “no” to the question, “Was your child given water or formula milk during the hospital stay?” Similarly, if the mother responded “yes” to “Did you receive breastfeeding counseling from doctors and nurses during your hospital stay?”, they were coded as receiving lactation counseling. Our fourth outcome, EBF at the time of survey, was based on whether respondents stated that their baby was currently exclusively consuming breastmilk, and not water or formula milk. The full survey, including these outcomes, can be seen in Appendix C.

### 2.3. Exposure and Covariates

The birth month of the infant and enrollment date of hospital were used to create a variable indicating if the hospital was enrolled at the time of each birth. Modality of birth (vaginal or cesarean) was collected in the survey. Birth months are summarized by quarters (spanning years 2019–2020) for descriptive purposes in Table 1.

### 2.4. Statistical Analysis

We used a segmented regression approach to measure the association of COE enrollment and the four main outcomes on the absolute risk difference (RD) scale [41]. Timing was defined in relation to hospital COE enrollment, ranging from five months before enrollment to five months after enrollment (dummy coded on a scale of −5 to 5, with month of enrollment coded as 0). We regressed each outcome for childbirth c in month m in hospital h on a centered indicator for month in relation to the time of enrollment, M, a binary indicator for enrollment (whether the child c was born in hospital h after it was enrolled in the COE program), E, and an interaction between month and enrollment using a linear probability model of the general form:

$$Y_{cmh}=\beta_{0}+\beta_{1}M_{m}+\beta_{2}E_{mh}+\beta_{3}M_{m}E_{mh}+\varepsilon_{cmh}$$

where $\beta_{1}$ estimates the linear pre-enrollment trend in the probability of the outcome, $\beta_{2}$ estimates the immediate change in the probability of the outcome after enrollment, and $\beta_{3}$ estimates the change from the pre-intervention trend after enrollment. We stratified models by modality of birth (vaginal/cesarean) to examine whether estimates differed by group. In all models, we included a random intercept for hospital to account for the clustering of births within hospitals. All analyses were executed in RStudio version 3.6.0, (RStudio, Boston, MA, USA) [42].

## 3. Results

### 3.1. Study Sample

Our study sample was derived from 28 hospitals spread across eight provinces throughout Vietnam (Appendix B). A total of 10,473 calls were made to households of infants recently born in these hospitals from January 2019 to April 2020. Of those contacted, 81% who answered the phone identified as the mother of the baby, with 10% a father, grandparent, or other caregiver who was able to answer the survey questions. The remaining 9% of phone calls were terminated due to the mother being unavailable, wrong number, or the baby had died. This resulted in 9585 respondents giving further information about the infant, with each hospital contributing between 2% and 7% of the births included in the final sample (Table 1).

Sample characteristics are presented in Table 1. Briefly, there was an even spread of infants born across each quarter of the study period, with 46% of births via cesarean. This reflects the oversampling of infants born via cesarean, which was by design—the national rate of cesarean births in Vietnam in 2019 was 33.6% [36]. All hospitals eventually enrolled in the COE program. After month six of the study period, only 6% of births surveyed were in un-enrolled hospitals.

**Figure 1:**
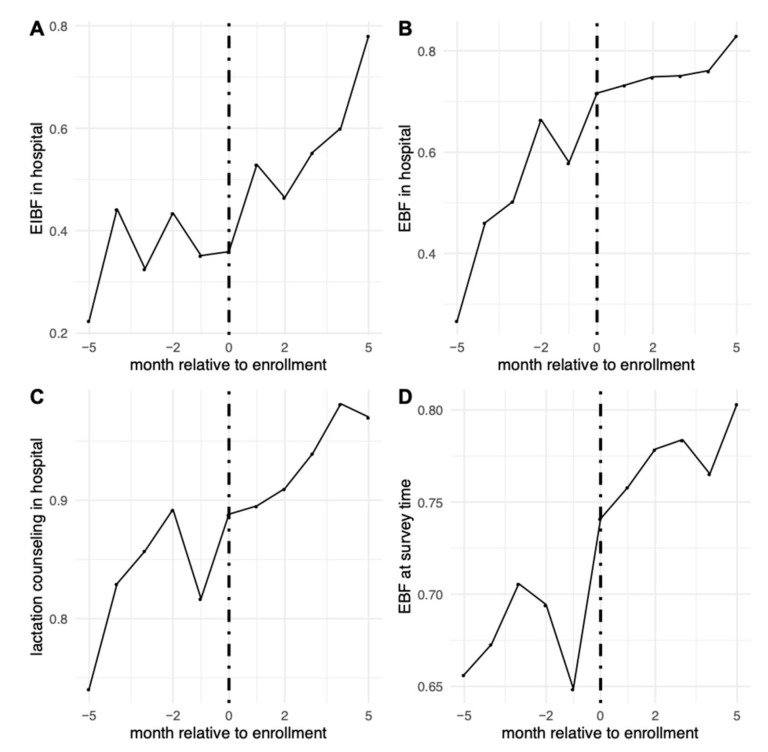
Rates of the main outcomes relative to timing of hospital enrollment. (**A**) Early initiation of breastfeeding in the hospital; (**B**) EBF in the hospital; (**C**) receiving lactation counseling in the hospital; (**D**) EBF at time of survey.

We examined rates of the four main outcomes among children born in hospitals before, during, and after enrollment (Figure 1). When looking at rates of the outcomes before hospitals enrolled, 33% reported EIBF, 53% reported that their newborn was EBF in the hospital, 84% reported receiving lactation counseling, and 69% reported that the infant was EBF at the time of the survey.

### 3.2. The Association between Enrollment and Breastfeeding Outcomes

Results from segmented regression models are shown in Table 2. Outcome trends before, during, and after enrollment can also be seen in Figure 1. Before enrollment, there were positive trends in the probabilities of EBF in hospital, which was increasing by roughly 3 percentage-points per month prior to COE enrollment. Enrollment was associated with an immediate increase in the levels of EIBF and EBF during hospital stay. After enrollment, there was an increase of 6 percentage-points (95% CI: 3, 9) in EIBF, with a post-enrollment increase in trend of 7 percentage-points (95% CI: 6, 8) per month. Enrollment increased the probability of EBF during hospital stay by 5 percentage-points (95% CI: 2, 9), after which the pre-intervention upward trend seemed to level off, with a post-enrollment decrease in trend of 2 percentage-points per month (95% CI: −3, −1). We did not observe evidence of an immediate association of enrollment with receipt of lactation counseling or EBF at survey time. However, the probability of lactation counseling, which was already highly prevalent in the pre-enrollment period, increased by 1 percentage-point per month (95% CI: 0, 2) in the post-enrollment period.

### 3.3. Variation by Modality of Birth

**Figure 2:**
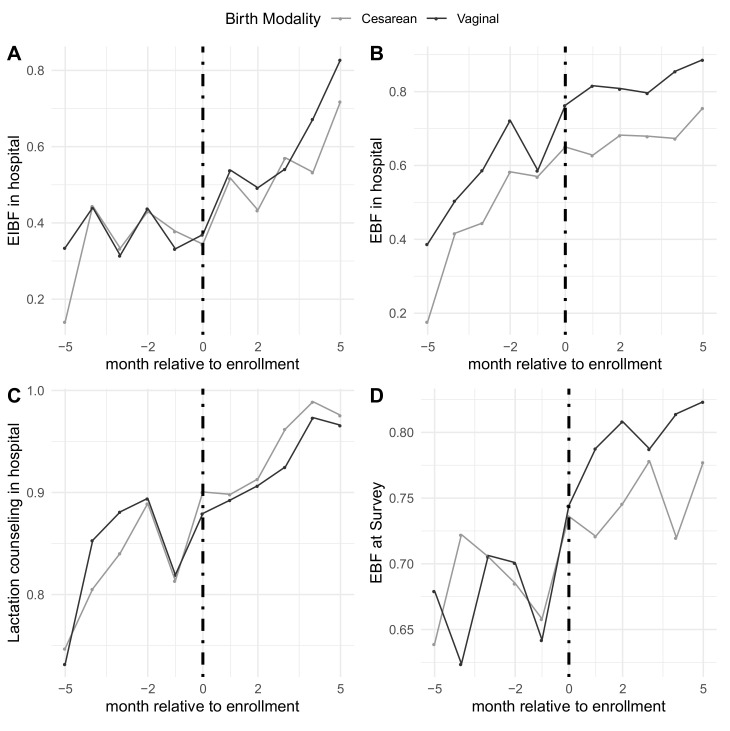
Rates of the main outcomes relative to timing of hospital enrollment separated by vaginal vs. cesarean births. (**A**) Early initiation of breastfeeding in the hospital; (**B**) EBF in the hospital; (**C**) receiving lactation counseling in the hospital; (**D**) EBF at time of survey.

Rates of the outcomes before, during, and after enrollment can also be seen in Figure 2. Pre-enrollment rates among women who gave birth via cesarean section were similar for all outcomes except EBF in hospital compared to women who gave birth vaginally; 31% vs. 35% for EIBF, 46% vs. 60% for EBF in hospital, and 83% vs. 84% for lactation counseling, respectively, and 69% for EBF at survey time for both. Patterns did not seem to vary by modality of birth, as shown in Table 3. Among respondents who gave birth vaginally, the pre-enrollment probability of EIBF was low with a flat pre-enrollment trend, enrollment was associated with an immediate increase of 4 percentage-points (95% CI: −0.2, 9) and an increasing, positive trend in the post-enrollment period of 9 percentage-points per month (95% CI: 7, 11). For EBF in both the hospital and at survey time, enrollment was associated with immediate increases of 6 (95% CI: 2, 10) and 4 (95% CI: −0.3, 8) percentage-points, respectively, respectively, with a flattening off in the post-enrollment trend. Similarly, among those who gave birth via cesarean section, trends for all outcomes were increasing slightly in the pre-enrollment period, enrollment was associated with immediate increases in EIBF and EBF during the hospital stay, and trends were similar in the post-enrollment period.

## 4. Discussion

We examined the association between enrollment in a novel baby-friendly hospital program and equitable support for breastfeeding in Vietnam using a segmented regression approach. There was evidence of positive trends in our primary outcomes even before hospitals received their improvement plans as part of the enrollment process, and an immediate and positive impact of enrollment on the probabilities of EIBF and EBF, both in the hospital and after discharge. Post-enrollment trends continued to increase for EIBF and lactation counseling and seemed to level off for both EBF in the hospital and at survey time. For both main and stratified analyses, we saw quite high baseline rates of lactation counseling, with relatively slight increases after enrollment. Additionally, while in both the main and stratified analyses we see slight decreases in the post-enrollment per month estimate, that appears to be a leveling-off effect that occurs later in the study period. While the strength of the enrollment and post-enrollment trends slightly varied, we observed similar effects of COE enrollment on the breastfeeding outcomes in both vaginal and cesarean births.

There are several limitations to this study. Firstly, all hospitals in this pilot study were eventually enrolled in the program, and we therefore do not have an external control group. Time-varying factors that might have influenced the timing of enrollment, as well as breastfeeding outcomes, could have introduced confounding bias [43]. Second, since hospitals were aware that they would be joining the COE program before the official enrollment date, defined by when the hospital had completed at least one training session, there may have been anticipatory effects. This could explain the positive outcome trends observed in the pre-intervention period. Third, mothers were interviewed in the newly postpartum period (between 10 and 45 days after discharge from the hospital), and due to having a newborn at home, it is possible there was recall bias when questioned about their time in the hospital. However, recall of EBF practices is an accepted and validated measurement of mothers within the breastfeeding literature [44], and we therefore do not anticipate it contributing to significant bias. Fourth, selection bias could have been introduced into the study. Selection of the analytic sample from the target population (hospitals enrolled during the study period) was not random, as hospitals were required to submit a minimum number of both vaginal and cesarean births to the surveyors. Because this program was being rolled out nationally, not all hospitals met the required number of cesarean births. If hospitals had a low number of cesarean births, they just submitted all of them to the surveyors. Additionally, while this study sample includes a large number of private and public hospitals from the eight provinces, the results of this study apply to this pilot study sample and should be generalized to all hospitals cautiously [45]. However, the number of private hospitals in this sample (14.29%) is reflective of the total number of private hospitals in Vietnam (14.26%) [46]. Finally, we acknowledge the possibility of confounding as there is evidence linking breastfeeding rates with maternal age and education [47,48]. However, because the intervention itself was at the hospital level, individual-level covariates are unlikely to affect the timing of enrollment of hospitals.

## 5. Conclusions

This study has found that infants who are born in hospitals after enrolling in the COE program were more likely to be exposed to better breastfeeding practices, particularly in EIBF. We also observed an immediate increase in EBF during the hospital stay, and an increasing trend in receiving lactation counseling in the post-enrollment period. Moreover, our findings indicate that enrollment in the COE program was associated with similar improvements in EIBF and EBF during the hospital stay for vaginal and cesarean births. For outcomes where pre-enrollment coverage was lower among cesarean births, such as EBF during the hospital study, the program therefore has the potential to reduce relative measures of health inequality and increase long-term infant and child health outcomes. The COE program is ongoing and scheduled to expand beyond the pilot sample of 28 hospitals utilized for these analyses. Future efforts to rigorously evaluate the program in a larger, representative sample are warranted.

## Figures and Tables

**Table 1:** Demographic characteristics.

Variables	*n*	%
Response *		
Mothers agreed to continue the call	8501	81.2%
Father, grandparents, or other caregiver agreed to continue the call	1084	10.4%
Mother refused to continue the call (stopped the call)	33	0.3%
Mother is busy (stopped the call)	134	1.3%
Phone number is wrong or not available (stopped the call)	703	6.7%
Infant died (stop the call)	18	0.2%
Birth Year and Quarter		
2019 Q1	1599	16.7%
2019 Q2	2125	22.2%
2019 Q3	1920	20.0%
2019 Q4	1951	20.4%
2020 Q1	1989	20.8%
Sex		
Male	3909	51.6%
Female	3637	48.0%
Twins	31	0.4%
Childbirth		
Vaginal Birth	5184	54.1%
Caesarian Birth	4401	45.9%
Term		
Pre-term (<37 weeks)	197	3.9%
Full term (≥37 weeks)	4888	96.1%
Private vs. Public Hospital		
Private	1039	10.8%
Public	8546	89.2%

* This breakdown includes every call made by the interviewer. The rest of the table only includes those (mothers or other caregivers) who agreed to participate.

**Table 2:** Risk difference from segmented regression models.

	EIBF	EBF During Hospital Stay	Lactation Counseling	EBF at Survey Time
	RD	RD	RD	RD
	(95% CI)	(95% CI)	(95% CI)	(95% CI)
Constant	*n = 9190*	*n = 9584*	*n = 9584*	*n = 9244*
	0.31	0.67	0.87	0.715
	(0.25, 0.38)	(0.60, 0.74)	(0.85, 0.90)	(0.68, 0.75)
Month	0.001	0.03	0.008	0.01
	(−0.01, 0.01)	(0.02, 0.04)	(0.002, 0.01)	(0.003, 0.02)
Enrolled	0.06	0.05	0.004	0.03
	(0.03, 0.09)	(0.02, 0.09)	(−0.02, 0.03)	(−0.004, 0.06)
Month × Enrolled	0.07	−0.02	0.009	−0.001
	(0.06, 0.08)	(−0.03, −0.005)	(0.002, 0.02)	(−0.01, 0.01)

The “×” signifies the multiplicaiton of Month and Enrollment.

**Table 3:** Risk difference from segmented regression models stratified by birth modality.

	EIBF	EBF During Hospital Stay	Lactation Counseling	EBF at Survey Time
	RD	RD	RD	RD
	(95% CI)	(95% CI)	(95% CI)	(95% CI)
*Vaginal Births*	*n = 5048*	*n = 5183*	*n = 5183*	*n = 5001*
Constant	0.33	0.71	0.88	0.71
	(0.26, 0.39)	(0.65, 0.77)	(0.85, 0.91)	(0.67, 0.75)
Month	−0.002	0.04	0.01	0.01
	(−0.02, 0.01)	(0.02, 0.05)	(0.003, 0.02)	(−0.001, 0.03)
Enrolled	0.04	0.06	−0.02	0.04
	(−0.002, 0.09)	(0.02, 0.01)	(−0.05, 0.01)	(−0.003, 0.08)
Month*Enrolled	0.09	−0.02	0.01	0.001
	(0.07, 0.11)	(−0.03, −0.005)	(−0.002, 0.02)	(−0.01, 0.02)
*Cesarean Births*	*n = 4142*	*n = 4401*	*n = 4401*	*n = 4243*
Constant	0.29	0.59	0.86	0.71
	(0.19, 0.38)	(0.51. 0.68)	(0.83, 0.89)	(0.66, 0.75)
Month	0.01	0.02	0.01	0.01
	(−0.01, 0.02)	(0.01, 0.04)	(−0.003, 0.01)	(−0.0001, 0.03)
Enrolled	0.08	0.05	0.03	0.01
	(0.02, 0.13)	(−0.003, 0.10)	(−0.002, 0.06)	(−0.04. 0.06)
Month*Enrolled	0.04	−0.01	0.01	−0.003
	(0.03, 0.06)	(−0.03, 0.004)	(−0.0002, 0.02)	(−0.02, 0.01)

## Data Availability

Deidentified and cleaned data, along with analytic code, can be found on the authors’ dataverse (https://dataverse.harvard.edu/dataverse/3po accessed on 18 June 2021) after publication of the article.

## Appendix A. Detailed Accreditation Process

In order to achieve accreditation, a hospital has to achieve Grade 4 on Criterion E1.3, as stipulated by the National Hospital Quality Standards [49], pass an external assessment, and receive positive feedback from the phone surveys with mothers after hospital discharge.

The external assessment is conducted by a five-member team, including two representatives from the MOH and/or provincial DOH, one CDC representative, and two independent experts on EENC and breastfeeding from the Learning & Research Center for Newborn Care and Human Milk. The purpose of the assessment is to provide an objective evaluation of maternal and early newborn care provided before, during, and after childbirth using eight checklists that measure adherence to BFHI and EENC standards, based on WHO’s Annual Implementation Review and Planning Guide [50], and the Vietnam MOH’s technical guidelines on breastfeeding, reproductive health [51], and EENC [52,53]. This included interviews with pregnant women; observations of pre-birth preparation, childbirth, and immediate postpartum activities for scheduled and emergency cesarean and vaginal childbirths; interviews with post-partum mothers for full-term and pre-term/low birth weight newborns; and assessment of hospital quality criteria and the breastfeeding enabling environment.

The MOH, Provincial DOH, or CDC are in charge of conducting the phone survey with mothers after hospital discharge. Every quarter, hospitals send the list of phone numbers of all discharged mothers (from the preceding quarter). This list is then separated into vaginal and cesarean childbirth. For hospitals not yet recognized as Centers of Excellence for Breastfeeding, the required sample size in a quarter is 100 successful surveys for provincial hospitals (50 vaginal deliveries, 50 cesarean deliveries), whilst district hospitals are required to complete 50 successful surveys (25 vaginal births, 25 cesarean births). Surveys are conducted on KoboToolbox platform server. Alive & Thrive randomly selected 5% of mothers who were previously surveyed and re-surveyed them for quality assurance. After the results are cross-checked, they are sent to hospitals quarterly for improvement plan and to DOH and MOH for monitoring.

For accreditation, hospitals were required to achieve a passing grade on all eight checklists and phone survey with mothers according to predetermined thresholds [54].

## Appendix B. Enrollment Date and Number of Births Sampled Per Hospital

**Hospital**	**n (Births)**	**% (Total Births in Sample)**	**Hospital Enrollment Date (Day/Month/Year)**
Ca Mau Obstetric and Pediatric Hospital	486	5.1%	2 April 2019
Tran Van Thoi General Hospital	294	3.1%	1 April 2019
Dam Doi General Hospital	222	2.3%	3 April 2019
U Minh District Hospital	248	2.6%	4 April 2019
Cai Nuoc General Hospital	252	2.6%	5 April 2019
Phu Vang District Hospital	259	2.7%	24 March 2019
Nam Dong District Hospital	249	2.6%	25 March 2019
Phong Dien District Hospital	250	2.6%	26 March 2019
Quang Dien District Hospital	233	2.4%	27 March 2019
Quang Nam Provincial General Hospital	580	6.1%	13 April 2019
Dong Giang District Hospital	235	2.5%	16 March 2019
Que Son District Hospital	228	2.4%	17 March 2019
Vinh Duc General Hospital	249	2.6%	16 March 2019
Quang Nam Central General Hospital	505	5.3%	15 March 2019
Quang Nam Regional General Hospital	495	5.2%	14 March 2019
Minh Thien General Hospital	260	2.7%	15 March 2019
Da Nang Hospital for Women and Children	508	5.3%	7 June 2019
Da Nang Family Hospital	252	2.6%	16 May 2019
Cam Le District Hospital	249	2.6%	16 May 2019
Son Tra District Hospital	252	2.6%	17 May 2019
Hai Chau District Hospital	243	2.5%	17 May 2019
Central Highland Region General Hospital	521	5.4%	11 February 2020
Cu Mgar District Hospital	255	2.7%	10 February 2020
Can Tho Obstetrics and Gynecology Hospital	503	5.2%	26 July 2019
Phuong Chau International General Hospital	278	2.9%	25 July 2019
Tu Du Hospital	502	5.2%	19 July 2019
Hung Vuong Hospital	507	5.3%	18 July 2019
Quang Ninh Obstetrics and Pediatrics Hospital	470	4.9%	24 May 2019
TOTAL	9585	100%	

## Appendix C. Survey Questionnaire

**No.**	**Question**	**Answer**
0	Call response	(1) Mother agreed to continue the call (moved to question 1) (2) Babysitter (father, grandparents) agreed to continue the call (moved to question 1) (3) Mother refuse to continue the call (stop the call) (4) Mother is busy (stop the call) (5) Phone number is wrong or not available (stop the call) (7) Baby died (stop the call)
1	How old is your youngest child?	(Note the child’s age in months)
1a	Is your child a boy or a girl?	(1) Boy (2) Girl (9) Others (twins, etc.)
2	Is s/he breastfed?	(1) Yes (move to question 2a) (2) No (move to question 3)
2a	Apart from breastfeeding your child, do you let your child eat or drink anything else?	(1) Exclusive breastmilk (from the biological mother or others) (2) Breastmilk and formula milk (3) Breastmilk and water (9) Breastmilk and other drinks/foods
3	Did you deliver him/her vaginally or via cesarean?	(1) Vaginally (2) Via cesarean
3a	Is your baby pre-term?	(1) <37 weeks (<259 days) (2) ≥37 weeks (≥259 days)
4	Was s/he placed on your chest/abdomen for skin-to-skin contact immediately after birth?	(1) Yes (move to question 5a) (2) No (move to question 5b) (8) No answer (9) Don’t know/don’t remember
5a	How long was s/he in skin-to-skin contact with you?	(1) Less than 90 min (2) More than 90 min (9) Don’t know/don’t remember
5b	How long after birth was your child returned to stay with you?	(1) Immediately or less than one hour (2) From one to six hours (3) More than six hours (9) Don’t know/don’t remember
6	Was your child breastfed within 90 min after birth?	(1) Yes (0) No (8) No answer (9) Don’t know/don’t remember
7	Was your child given water or formula milk during the hospital stay?	(1) Yes (0) No (8) No answer (9) Don’t know/don’t remember
8	Did you receive breastfeeding counseling from doctors and nurses during your hospital stay?	(1) Yes (0) No (8) No answer (9) Don’t know/don’t remember
9	Did doctors and nurses in the hospital counsel you to use formula milk for babies aged under 24 months?	(1) Yes (move to question 9a) (0) No (8) No answer (9) Don’t know/don’t remember
9a	Why were you counseled to use formula milk by doctors/nurses?	(1) Having little breastmilk/no breastmilk (2) Cesarean childbirth (3) The mother is sick, thus not able to breastfeed her baby (4) The baby is sick or born preterm, thus not able to breastfeed (5) The mother wants to feed her baby with formula milk (7) Other (please specify) (8) No answer (9) Don’t know/don’t remember
10	Did you see any forms of advertising/marketing of formula milk for babies aged under 24 months, feeding bottles and artificial pacifiers in the hospital?	(1) Posters or advertisements of infant formula for babies aged under 24 months (2) Formula company staff marketing formula milk for children under 24 months at the hospital (3) Formula milk products for children under 24 months being displayed for sales or introduced by health staff (4) Persons asking for your phone number and calling you to introduce breastmilk substitutes after birth, feeding bottles, or artificial pacifiers (5) Advertisements of formula milk for pregnant women and postpartum mothers (6) Feeding bottles and artificial pacifiers being advertised and displayed for sale (9) Other types of advertisements/marketing about formula milk for babies aged less than 24 months (describe) (0) No abovementioned forms seen
11	Did you have a birth companion of choice at the childbirth ward?	(1) Yes (0) No
12	What is your ethnicity?	(1) Kinh (2) Tay (3) Thai (4) Hoa (5) Khmer … (56) Other (specify in question 12a)
12a	Please specify your ethnicity?	
13	Do you have any recommendations for the hospital to better support breastfeeding?	(1) Yes (note down the comments) (0) No
